# Gestational Selenium Exposure and Neonatal Health Outcomes: A Systematic Review of Mortality, Morbidity, and Neurobehavioral Development

**DOI:** 10.3390/jcm15166255

**Published:** 2026-08-13

**Authors:** Nikolina Stachika, Ermioni Tsarna, Eleni Solomou, Stavroula-Ioanna Kyriakou, Sofoklis Stavros, Panagiotis Christopoulos

**Affiliations:** 1 Second Department of Obstetrics and Gynecology, “Aretaieion” University Hospital, Faculty of Medicine, National and Kapodistrian University of Athens, 11528 Athens, Greece; nnikolinast@gmail.com (N.S.); ermioni.tsarna@gmail.com (E.T.);; 2 Third Department of Obstetrics and Gynecology, General University Hospital Attikon, Faculty of Medicine, National and Kapodistrian University of Athens, 12462 Athens, Greece

**Keywords:** selenium, pregnancy, neonatal mortality, neonatal morbidity, neonatal neurodevelopment

## Abstract

**Objectives**: This systematic review synthesized the existing literature to evaluate the associations between maternal Selenium (Se) exposure during pregnancy and neonatal outcomes, specifically focusing on parameters of mortality, morbidity, and neurobehavioral development. **Methods**: PubMed, Embase, and the Cochrane Library were systematically searched from inception through December 2025. For quality appraisal, Cochrane RoB2 and the National Heart, Lung, and Blood Institute of the National Institutes of Health tools for observational studies were applied. Results were qualitatively synthesized. **Results**: Screening of 2743 unique records and 473 full-text articles yielded 355 papers included in the overarching SeduP project, with 19 specific to this review. Synthesized evidence revealed a lack of consistent evidence of an association between maternal Se status and all-cause neonatal or perinatal mortality (*n* = 413, 4 studies), combined mortality/morbidity (*n* = 2753, 2 studies), or infectious-related morbidity (*n* = 928, 3 studies). Conversely, conflicting findings were observed regarding all-cause neonatal morbidity (*n* = 68, 2 studies) and neurobehavioral development (*n* = 1442, 4 studies), while a potentially inverse association was identified exclusively between Se levels and pulmonary-related neonatal morbidity (*n* = 611, 4 studies). **Conclusions:** Despite compelling preclinical biological plausibility, clinical data do not support a widespread protective effect of maternal Se status against adverse neonatal outcomes. The existing body of evidence is heavily constrained by moderate to very low quality, driven by small sample sizes, insufficient confounding adjustments, and suboptimal exposure assessment timing at delivery. While localized signals imply potential pulmonary protection alongside risks of excess toxicity, large-scale, standardized prospective cohorts are critically needed to generate clinically actionable data.

## 1. Introduction

Neonatal mortality, defined as infant death occurring within the first 28 days of life, accounts for a striking 47% of all deaths among children under five years of age globally [1]. These mortality rates exhibit profound disparities across geographic regions and socioeconomic gradients, with African regions bearing the heaviest epidemiological burden [2,3,4]. Concurrently, neonatal morbidity, particularly among preterm and low-birth-weight infants, frequently necessitates admission to the neonatal intensive care unit (NICU) and is predominantly characterized by perinatal infections and respiratory distress syndrome (RDS), alongside underlying congenital anomalies, while long-term neurobehavioral issues are also noted [5,6]. Despite the remarkable technological and clinical advancements in neonatal medicine over the past decades, neonatal mortality and morbidity persist as critical global public health challenges. Consequently, research continues to investigate modifiable risk factors, utilizing the gestational period as a crucial window of opportunity for clinical and/or public health intervention. Within this context, studies have explored a wide range of maternal determinants, including environmental and occupational exposures, lifestyle factors, physical activity, and nutritional status, alongside systemic healthcare access and socioeconomic equity.

Selenium (Se) is an essential trace element that is primarily obtained through dietary intake, which consequently varies widely across global populations. Meat, poultry, grains, and cereals generally constitute the primary dietary sources of Se, although seafood serves as the predominant source in specific coastal or island populations [7,8]. At the population level, the geographic region of residence plays a deterministic role in systemic exposure, driven heavily by regional variations in soil Se concentrations [9]. To maintain optimal blood Se levels of 80–140 ng/mL, the recommended daily Se intake ranges from 26 μg to 34 μg depending on sex [7]. The clinical significance of Se in human health and disease has been extensively investigated, with established roles documented in thyroid function, neuroprotection, immune system regulation, fertility, diabetes, cardiovascular disease, oncology, and overall mortality [8]. Both Se deficiency and excess have been linked to neurological disorders; furthermore, deficiency states can precipitate cardiovascular, endocrine, and immunological manifestations, whereas selenium toxicity predominantly impacts the gastrointestinal and reproductive systems [7,8,9,10,11,12,13]. Chronic selenosis can additionally manifest clinically with anemia, fatigue, depression, nail dystrophies, alopecia, and a characteristic garlic-like breath odor [7,9].

With respect to the gestational period, Se exerts vital antioxidant properties through its structural integration into functional selenoproteins, which are crucial for normal fetal and infant development. Nevertheless, maternal Se concentrations frequently decline over the course of gestation, driven by enhanced metabolic demands for oxygen transport and robust antioxidative protection within the rapidly developing fetoplacental unit [14]. Notably, the prevalence of gestational Se deficiency is highly variable on a global scale, ranging from being an exceedingly rare condition in the USA to being almost universal in countries like Zambia [15,16]. Within European regions, a high prevalence of gestational Se deficiency has also been documented, reaching up to 79% in a Polish cohort [17].

Taking into consideration the public health relevance of identifying modifiable risk factors for neonatal mortality, morbidity, and neurodevelopment, paired with the considerable prevalence of Se deficiency during pregnancy, even a modest causal association between maternal antenatal Se exposure and these infant outcomes could have important public health implications. Therefore, the aim of this systematic review was to systematically search and synthesize the existing literature to elucidate the potential associations between maternal Se exposure during pregnancy and early health trajectories of the newborn, operationalized through parameters of mortality, morbidity, and neurobehavioral development.

## 2. Materials and Methods

The primary objective of the Selenium Exposure during Pregnancy (SEduP) Project is to systematically gather and critically appraise the global literature investigating the relationship between gestational Se status and subsequent maternal, fetal, and neonatal outcomes. For the overarching project, relevant outcomes encompass any maternal and/or fetal clinical outcomes manifesting throughout gestation or during the immediate four-week postpartum period. The specific systematic review presented herein, however, isolates and analyzes only the subset of data directly characterizing neonatal and perinatal mortality and neonatal morbidity. Within this framework, neonatal mortality is defined as infant death occurring within the first 28 days post-delivery, while perinatal mortality additionally includes stillbirths. Morbidity comprises early-life complications, such as respiratory distress syndrome and perinatal infections, alongside early indicators of long-term neurodevelopmental sequelae, all assessed within the first four weeks of life.

Eligibility was restricted to original, peer-reviewed research disseminated as full-text journal articles and original research disseminated as conference posters, representing gray literature. Methodologically, the review considered randomized controlled trials (RCTs), prospective cohorts, cross-sectional analyses, and case–control designs that either evaluated the clinical impact of antenatal Se supplementation or quantified Se concentrations in biological matrices (including maternal blood or urine, umbilical cord blood, and placental tissue) relative to the specified infant outcomes. Conversely, several exclusion criteria were applied. Studies were excluded if they were published in languages other than English, if the full-text manuscript proved unretrievable, or if they represented case reports or non-original publication types, such as narrative reviews, editorials, systematic reviews, meta-analyses, or letters to the editor. Furthermore, investigations evaluating multi-nutrient or multivitamin regimens where the independent effect of selenium could not be isolated were disqualified, as were studies tracking infant outcomes exclusively beyond the initial four-week postnatal window.

To identify eligible literature, a comprehensive search strategy was developed across three electronic databases: PubMed, Embase, and the Cochrane Library, covering all literature indexed through December 2025. The core search algorithm was initially built for PubMed utilizing a combination of free-text keywords and Medical Subject Headings (MeSH) mapped to Se and gestation (Table 1). This baseline syntax was subsequently translated for Embase and the Cochrane Library using the Polyglot search tool [18] (Tables S1 and S2). To ensure a highly sensitive and exhaustive retrieval process aligned with the broader goals of the SEduP project, the search strings omitted specific terms for individual clinical outcomes, remaining intentionally generalized to capture all intersecting literature on pregnancy and Se.

Following the initial database search, all retrieved citations were uploaded into the Rayyan web application to facilitate deduplication and blind screening [19]. Two investigators (N.S. and S.-I.K.) independently evaluated the titles and abstracts of the unique records while remaining blinded to each other’s decisions, eliminating clearly irrelevant literature. Any screening conflicts at this stage were mediated through consensus and consultation with a senior investigator (E.T.). The full-text manuscripts of potentially eligible studies were subsequently retrieved and uploaded to Rayyan for a secondary round of screening. The same dual-investigator, blinded methodology was applied during the full-text review. Manuscripts were permanently included only if their experimental protocols strictly fulfilled all predefined eligibility criteria and their results were explicitly and independently reported. For organizational clarity, included studies were tagged according to their specific outcome parameters. To ensure literature saturation, the reference lists of all accepted articles were manually searched to capture any relevant studies missed by the electronic search algorithm. Finally, all included records within each outcome subgroup were cross-referenced to identify overlapping study populations by comparing authors, affiliated institutions, study periods, and geographic locations. In cases of overlapping cohorts, we selected either the record providing the most comprehensive methodology and outcome reporting or the record with the larger analytical sample size (e.g., publications after data collection completion vs. preliminary analyses).

To synthesize the essential evidence, study characteristics and primary outcomes were extracted and organized into a comprehensive Summary of Findings (SoF) matrix (Table S3). The initial extraction and tabulation were executed by one reviewer (N.S.), and the accuracy of the transcribed data was subsequently verified by two independent reviewers (E.S. and E.T.). The specific variables included within the SoF matrix comprised the following parameters: primary author and publication year, dissemination type (full-text research article or conference poster), geographic location of the cohort (including urban or rural designations, where available), epidemiological study design and sample size, modality of selenium evaluation (interventional supplementation or biomarker measurement), chronological timing of the selenium assessment, explicit selenium exposure levels or dosing regimens, classification of the investigated clinical endpoint, statistical methodology employed by the investigators, principal findings, and lastly a narrative summary of the study’s conclusions. With regard to study design and sample size, data extraction focused strictly on the final analytical sample size of mother–child pairs included in the exposure-outcome analyses rather than total baseline enrollment. Study designs were similarly operationalized based on the specific analytical framework used for the Se assessment rather than the overarching parent study design.

A formal quantitative meta-analysis was considered separately per outcome and was deemed inappropriate for the retrieved data. This decision was driven by the limited volume of qualifying studies and the substantial clinical and methodological heterogeneity regarding the reported neonatal endpoints and statistical modeling. Consequently, a qualitative synthesis was adopted. To structure the narrative analysis, the literature included was stratified into distinct thematic sub-categories based on the precise neonatal clinical outcome evaluated. These categories comprised all-cause neonatal or perinatal mortality, all-cause neonatal morbidity, combined neonatal mortality and morbidity, neonatal pulmonary conditions, namely Respiratory Distress Syndrome (RDS) and Bronchopulmonary Dysplasia (BPD), infectious drivers of neonatal morbidity and/or mortality, including neonatal sepsis alongside the intrauterine transmission of cytomegalovirus (CMV) and Human Immunodeficiency Virus (HIV), and lastly neurobehavioral development.

To evaluate the internal validity of the included literature, study-specific quality appraisal frameworks were implemented based on study design. RCTs were critically appraised using the Cochrane Risk of Bias 2 tool, applying a per-protocol analysis effect to evaluate the impact of adhering to the intervention [20] (Table S4). Observational designs, including cohort, cross-sectional, and case–control studies, were evaluated using the quality assessment framework developed by the National Heart, Lung, and Blood Institute of the National Institutes of Health [21] (Tables S5 and S6). While unstandardized, these tailored tools have been successfully implemented in prominent systematic reviews and were highly aligned with the specific structural characteristics of the current project. All tools’ questions were discussed in a group meeting to a priori achieve consensus for bias risk across different domains. In cohort designs, exposure quantification must have preceded outcome assessment to verify temporal precedence for potential causal relationships. Because cross-sectional analyses capture data concurrently, they were structurally disqualified from being rated as entirely free of bias in this temporal domain. Furthermore, given that Se status is a continuous biological variable, studies were required to model it continuously or stratify exposure levels into three or more hierarchical categories to achieve a low risk of bias score for exposure assessment. Finally, to minimize confounding bias within the statistical analyses, studies were required to incorporate crucial gestational and sociodemographic covariates into their multivariable models, such as maternal age, gestational age at birth, infant birth weight, antenatal smoking status, and parental socioeconomic or educational background. The described risk of bias assessment was executed independently by two investigators (N.S. and E.S.), with disagreements being resolved through consultation with a senior reviewer (E.T.).

To assess the certainty in the body of evidence, we utilized the Grading of Recommendations Assessment, Development, and Evaluation (GRADE) tool for exposures [22]. The assessment was conducted independently for every outcome subgroup, through consensus discussion by two reviewers (N.S. and E.T.). According to the GRADE methodology, both interventional (RCTs) and observational studies (Cohorts and Case–Control) are initially classified as high-certainty evidence. They are subsequently downgraded if they exhibit limitations in one or more of the five standard domains: risk of bias, inconsistency, indirectness, imprecision, and dissemination bias. Specifically, inconsistency was assigned if the included studies reported conflicting association shapes or opposing directions of effect. Indirectness was primarily evaluated based on the methodology used for exposure assessment; studies were downgraded if Se exposure was measured using umbilical cord blood or tissue, placental tissue, or maternal blood collected at the time of delivery or shortly postpartum. Secondarily, indirectness was evaluated based on the evaluation of clinically distinct outcomes within the same outcome subgroup. Ultimately, the certainty of evidence for each subgroup of outcomes was categorized into one of four levels: high, moderate, low, or very low.

The design, execution, and reporting architecture of this systematic review adhere to the updated Preferred Reporting Items for Systematic Reviews and Meta-Analyses (PRISMA) guidelines [23] (Tables S7 and S8).

## 3. Results

The initial electronic database search yielded a total of 4000 records, comprising 1449 citations from PubMed, 2308 from Embase, and 243 from the Cochrane Library, all of which were imported into Rayyan. Following the automated identification and manual removal of 1257 duplicate records, 2743 unique articles underwent title and abstract screening. Out of these, 499 records fulfilled the criteria for full-text evaluation, and their complete reports were subsequently sought. Due to indexing gaps within journal archives or a general lack of institutional accessibility, the full text of 26 articles could not be retrieved, leading to their exclusion.

**Figure 1:**
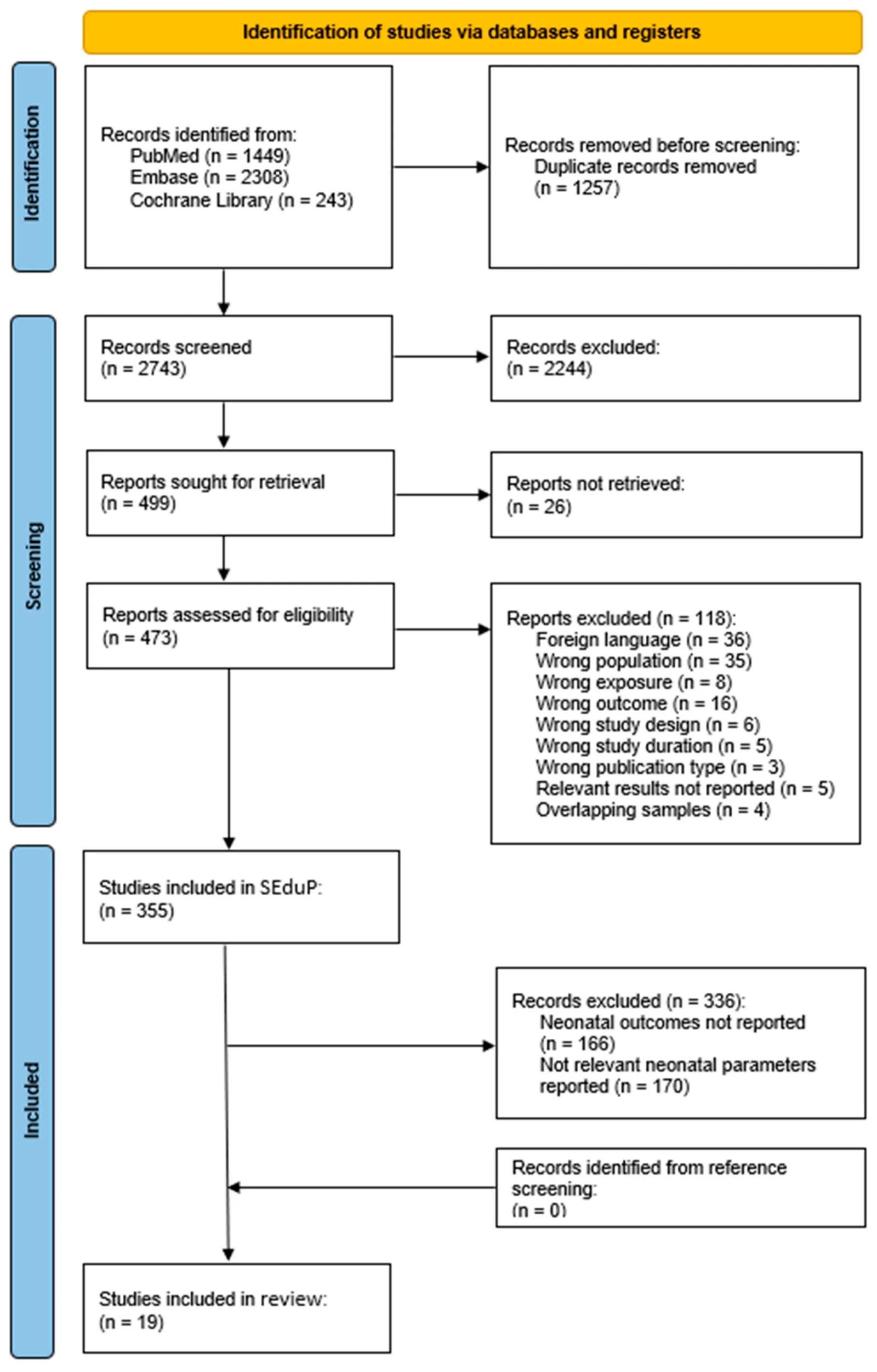
Flow Diagram of Studies Included in the Systematic Review.

The remaining 473 full-text manuscripts were screened against the eligibility criteria, resulting in the exclusion of 118 articles and leaving 355 studies within the broader SeDuP pool. The specific justifications for the full-text exclusions were as follows: 36 studies were published in languages other than English; 35 evaluated non-pregnant cohorts; 16 targeted irrelevant clinical endpoints; 8 did not include a Se assessment; 6 failed to measure Se effect independently; 5 tracked outcomes exclusively past the first month of postnatal life; 5 failed to adequately present relevant statistical data; and 3 represented ineligible publication types. Furthermore, 4 studies were excluded because they evaluated a patient population that was already represented in another included paper that reported the data more comprehensively. Out of 355 studies included in the overarching SEduP project, 166 examined solely pregnancy-related outcomes rather than neonatal outcomes, while 170 reported on neonatal parameters other than mortality, morbidity, or neurodevelopment, such as gestational age at birth, neonatal anthropometrics, congenital anomalies, vitamin D deficiency, and neonatal thyroid function. Ultimately, out of the overarching project portfolio, a distinct subset of 19 articles specifically evaluating neonatal neurodevelopment or conditions driving neonatal morbidity and mortality met all criteria and were selected for this systematic review. Secondary manual screening of the references of these eligible studies yielded no additional relevant records. This comprehensive selection process is visually mapped in the accompanying PRISMA flow diagram (Figure 1).

Among the 19 included studies, all-cause neonatal or perinatal mortality was investigated in four articles, capturing data from 413 mother–child pairs. All-cause neonatal morbidity was isolated in two studies featuring a combined sample of 68 mother–child pairs, while an additional two investigations concurrently examined both neonatal mortality and morbidity across a large pool of 2753 mother–child pairs. Specific neonatal morbidity secondary to pulmonary diseases was evaluated in four articles, utilizing clinical data extracted from 611 mother–child pairs. Infectious etiologies driving neonatal morbidity were the primary focus of three studies, which collectively spanned 928 mother–child pairs. Lastly, four studies targeted neonatal neurobehavioral development, representing an aggregated sample size of 1442 mother–child pairs.

### 3.1. All-Cause Neonatal or Perinatal Mortality

Among the four articles evaluating the prevalence of neonatal or perinatal mortality, three were prospective analyses derived from two cohorts [24,25] and one case–control study [26], while one was an RCT [27]. The evaluated study populations spanned four geographically distinct countries, Nigeria, Iran, India and Greece, focusing predominantly on urban settings. Crucially, the sample sizes across these analyses were notably small for the evaluation of mortality outcomes, ranging from only 51 to 180 participants and thus reflecting low statistical power. To be noted, two studies evaluated perinatal mortality, a composite outcome including both stillbirths and neonatal deaths [24,27]; data could not be extracted solely for neonatal mortality due to reporting limitations in these studies [24,27]. Ultimately, no study identified a statistically significant association between maternal Se status or antenatal Se supplementation and rates of perinatal or neonatal mortality.

Regarding exposure assessment, two studies quantified Se concentrations in maternal venous blood during the second or third trimester (OR = 1.29, 95% CI 0.03–5.97 [24] and hypothesis testing *p* > 0.05 [25]) [24,25], whereas one measured Se levels in umbilical cord blood at the time of delivery (OR = 1.16, 95% CI 0.85–1.58) [26]; all three yielded non-significant results. Notably, the latter study was conducted in a higher-risk population that included pregnancies complicated by pre-eclampsia and eclampsia alongside unaffected controls. Finally, in the only RCT, antenatal supplementation with 200 μg of Se daily throughout pregnancy failed to significantly alter neonatal mortality rates (Fisher’s test *p* = 0.932), though the statistical power was heavily constrained by a small sample and low event rate (*n* = 180, 4 perinatal deaths) [27].

In conclusion, the currently available clinical and epidemiological data do not support a protective effect of maternal Se exposure during pregnancy against neonatal death. However, this definitive null finding must be interpreted with caution; statistical power was presumably inadequate across all analyses and adjustment for potential confounding variables was entirely absent or inadequately reported in all four articles.

### 3.2. All-Cause Neonatal Morbidity

Two studies evaluated parameters of neonatal morbidity as composite outcomes, although their operational definitions of this endpoint diverged. In one study, morbidity was defined as respiratory distress syndrome (RDS) and admission to the neonatal intensive care unit (NICU) [28], whereas in the other as neonatal hyperbilirubinemia and overall hospitalization rates [29]. Methodologically, one was an RCT [29] and the other a prospective cohort study [28]. The RCT, conducted in Iran, evaluated the impact of a daily 200 μg Se supplementation regimen vs. placebo among 36 pregnant individuals diagnosed with gestational diabetes mellitus (GDM) [29]. Conversely, the prospective cohort study, carried out in Georgia, quantified Se concentrations within both maternal venous and umbilical cord blood matrices across 32 mother–child pairs [28].

The findings from these two investigations yielded contrasting results regarding the clinical impact of Se exposure. Results from the interventional trial indicated that antenatal Se supplementation in GDM-affected pregnancies significantly lowered the risk of neonatal morbidity (Chi-square test *p* = 0.03) [29]. In direct contrast, the observational cohort data revealed an adverse association, wherein elevated Se concentrations in maternal blood were correlated with an increased risk of neonatal RDS (r = 0.36, *p* < 0.01) and higher rates of NICU referrals (r = 0.50, *p* < 0.01) [28]. Notably, the interpretability of both datasets is heavily constrained; neither study performed statistical adjustments for critical confounding variables, yet no apparent randomization issues were identified in the RCT, and the sample sizes in both instances were strikingly small, leaving them highly susceptible to type I and type II errors.

### 3.3. Combined Neonatal Mortality and Morbidity

Two studies evaluated composite endpoints capturing concurrent rates of neonatal mortality and morbidity [30,31]. In an Iranian RCT investigating an antenatal regimen of 100 μg of Se administered daily from the first trimester to delivery, no statistically significant effect on composite neonatal mortality or morbidity risks was observed (*n* = 125) [30]. Conversely, a large-scale Norwegian prospective cohort study involving 2628 mother–child pairs demonstrated that while maternal blood Se concentrations were not associated with the primary composite risk of neonatal mortality and morbidity (unadjusted = 1.10, 95% CI 0.90–1.30, adjusted OR = 0.84, 95% CI 0.84–1.25), they were significantly and positively correlated with a higher risk for neonatal interventions in the unadjusted analysis (OR = 1.12, 95% CI 1.01–1.25) [31]. Within that cohort, these interventions were operationally defined as transfer to the NICU, requirement for mechanical ventilation or continuous positive airway pressure (CPAP) support, or administration of systemic antibiotic therapy. Notably, upon multi-variable adjustment for relevant confounding variables, statistical significance was lost, despite the magnitude of the effect size remaining reasonably stable (OR = 1.11, 95% CI 0.99–1.24).

In conclusion, the currently available literature does not support a protective effect of maternal antenatal Se exposure against composite neonatal mortality and morbidity. While an initial association of higher Se levels with increased neonatal interventions was observed in a large Norwegian cohort, this relationship became statistically non-significant after multivariable adjustment, indicating substantial statistical uncertainty that warrants cautious interpretation and further replication.

### 3.4. Neonatal Pulmonary Conditions

Neonatal morbidity secondary to pulmonary conditions among preterm neonates was evaluated in relation to Se exposure across four studies conducted in Iran, New Zealand, and the USA [32,33,34,35]. Within this literature, two small cohort studies [33,34] and one case–control study [32] specifically investigated bronchopulmonary dysplasia (BPD) and/or respiratory distress syndrome (RDS). In contrast, a considerably larger cohort study comprising 457 mother–child pairs examined oxygen dependency at 28 days post-delivery as its primary pulmonary endpoint [35]. Regarding exposure matrix sampling, only the largest cohort quantified Se concentrations in maternal blood [35], whereas the remaining three studies measured Se levels in umbilical cord blood [32,33,34]; all biological samples across these studies were collected at the time of delivery.

The highly powered New Zealand cohort reported that higher maternal Se levels were significantly associated with lower neonatal oxygen dependency at 28 days, an association that remained statistically significant after adjusting for potential confounding variables (OR = 0.79, 95% CI 0.62–0.99) [35]. Aligning with these findings, the American case–control study observed significantly lower umbilical cord Se concentrations among neonates diagnosed with BPD or RDS compared to healthy controls, though this finding was limited by an unadjusted statistical analysis (ANOVA *p* < 0.05) [32]. Conversely, the two smaller Iranian cohorts, with sample sizes ranging from only 19 to 54 dyads, failed to detect any statistically significant associations between cord Se status and the development of RDS or BPD in similarly unadjusted analyses (Student’s t test *p* = 0.17 [33] and Student’s t test *p* = 0.68 [34]) [33,34].

In conclusion, epidemiological data suggest an inverse association between adequate Se levels and the risk of neonatal pulmonary morbidity. However, important methodological limitations warrant caution when interpreting these data. All available studies quantified Se status at the time of parturition rather than longitudinally during the antenatal period, and the maternal compartment was directly sampled in only a single investigation.

### 3.5. Infectious Drivers of Neonatal Mortality and/or Morbidity

Three prospective cohort studies have to date evaluated infectious drivers of neonatal morbidity, although their examined clinical outcomes diverged significantly [36,37,38]. These endpoints included the incidence of neonatal sepsis among preterm infants (Q1: reference, Q2: HR = 0.92, 95% CI 0.45–1.88, Q3: HR = 0.82, 95% CI 0.36–1.90, Q4: HR = 0.60, 95% CI 0.28–1.29, *p* = 0.16) [36], intrauterine cytomegalovirus (CMV) transmission among pregnancies complicated by CMV infection (Student’s t test *p* > 0.05) [38], and vertical, intrauterine HIV transmission (binomial regression analysis *p* = 0.25) [37]. Geographically, these investigations spanned distinct socioeconomic environments, including the USA [36], rural regions of China [38], and an urban region of Tanzania [37]. Ultimately, none of the studies detected a statistically significant association between Se concentrations in either maternal or umbilical cord blood and the aforementioned infectious outcomes.

Statistical power and methodological approaches varied considerably across these studies. The investigation into intrauterine CMV transmission was likely underpowered due to its limited sample size (*n* = 48) and was notably the only study that relied on an unadjusted statistical analysis [38]. In contrast, the cohort studies evaluating neonatal sepsis (*n* = 269) [36] and intrauterine HIV transmission (*n* = 611) [37] were substantially better powered and deployed multivariable models to account for potential confounding variables.

In conclusion, current prospective evidence does not indicate that maternal Se status independently influences vertical viral transmission rates or early-life bacterial sepsis susceptibility. However, the sparse distribution of data across distinct clinical outcomes limits the ability to draw generalized conclusions regarding the broader role of Se in neonatal immunology.

### 3.6. Neonatal Neurobehavioral Development

Four prospective cohort studies evaluated neonatal neurobehavioral development across diverse geographic settings, including the USA, China, and the Faroe Islands, capturing both urban and rural populations [39,40,41,42]. Cohort sample sizes ranged from 141 to 927 mother–child pairs. Across all investigations, Se status was quantified exclusively at the time of delivery using indirect biological matrices, namely umbilical cord blood [39,41] or placental tissue [40,42], serving as a proxy for late-gestational exposure. Infant neurobehavioral outcomes were evaluated within the first two weeks of life utilizing three distinct, validated instruments, namely the Neonatal Behavioral Neurological Assessment (NBNA) [39], the Neonatal Intensive Care Unit Network Neurobehavioral Scale (NNNS) [40,42], and the Neurologic Optimality Score (NOS) [41]. These metrics represent widely accepted, scientifically robust tools designed to systematically characterize multiple domains of early neurological maturation and neonatal behavior during the first four weeks of life [41,43,44,45].

With respect to cord blood Se, an inverted U-shaped non-linear association was identified in a large Chinese cohort of 927 mother–child pairs [39]. Within this population, both low and high cord blood Se concentrations correlated with depressed overall NBNA scores (for Se < 100 μg/L: β = 4.4, 95% CI 3.6–5.2, *p* < 0.001; for Se ≥ 100 μg/L: β = −3.6, 95% CI −6.2–1.1, *p* = 0.006). Analysis of the NBNA subscales revealed that this adverse effect was driven by impairments in the behavioral (11.6 ± 0.8 for Se < 100 μg/L vs. 11.9 ± 0.5 for Se ≥ 100 μg/L, *p* < 0.001) and passive muscle tone domains (7.6 ± 0.7 for Se < 100 μg/L vs. 7.9 ± 0.4 for Se ≥ 100 μg/L, *p* < 0.001), whereas active muscle tone (7.9 ± 0.3 for Se < 100 μg/L vs. 8.0 ± 0.2 for Se ≥ 100 μg/L, *p* > 0.05), primitive reflexes (6.0 ± 0.1 for Se < 100 μg/L vs. 6.0 ± 0.2 for Se ≥ 100 μg/L, *p* > 0.05), and general activity scores (6.0 ± 0.2 for Se < 100 μg/L vs. 6.0 ± 0.1 for Se ≥ 100 μg/L, *p* > 0.05) remained unaffected [39]. Conversely, a smaller study involving 182 pairs from the Faroe Islands identified no statistically significant correlation between cord blood Se and neurological development (Spearman’s correlation r = 0.06, *p* > 0.05) [41]. Notably, in contrast to the multi-variable adjustments deployed in the Chinese study, the Faroese analysis reported only unadjusted analysis, exploring the linear association between cord blood Se concentrations and the aggregate NOS.

Regarding the placental tissue Se, two American studies with comparable sample sizes (*n* = 141 and *n* = 192 mother–child pairs) employed adjusted multivariable models to examine the relationship between placental Se content and NNNS profiles, yet they yielded divergent conclusions [40,42]. While one study observed no significant neurological alterations (*p* > 0.05, OR and 95% CI not reported) [42], the other reported that increasing placental Se concentrations were significantly associated with decreased muscle tone across the neonatal trunk and extremities (OR = 1.76, 95% CI 1.12–2.82), paired with heightened infant excitability (β = 0.76, standard error = 0.35, *p* = 0.03) [40]. However, other discrete NNNS subscales, including attention (β = 0.29, standard error = 0.22, *p* = 0.19), quality of movement (β = 0.003, standard error = 0.19, *p* = 0.99), self-regulation (β = −0.18, standard error = 0.20, *p* = 0.39), habituation (β = −0.61, standard error = 0.59, *p* = 0.31), stress/abstinence signs (β = −0.004, standard error = 0.01, *p* = 0.62), arousal (β = 0.19, standard error = 0.19, *p* = 0.30), handling characteristics (β = −0.02, standard error = 0.03, *p* = 0.40), non-optimal reflexes (β = −0.31, standard error = 0.25, *p* = 0.21), lethargy (β = 0.10, standard error = 0.30, *p* = 0.73), hypertonicity (OR = 1.04, 95% CI 0.62–1.71), and asymmetric reflexes (OR = 1.03, 95% CI 0.68–1.57), did not vary across the exposure gradient [40].

In summary, no study to date has directly quantified maternal Se concentrations longitudinally during the pregnancy, with all literature relying on matrices collected at birth. While there are isolated epidemiological indications pointing toward a potential negative impact of elevated Se exposure on neonatal muscle tone, this finding was not reproducible across similar cohorts, and changes were restricted to specific localized subscales rather than comprehensive primary scores. Consequently, the statistical robustness of this adverse association remains highly questionable, an underlying causal framework is far from established, and clinical relevance is, thus, questionable.

### 3.7. Quality Appraisal of the Included Studies

Regarding neonatal or perinatal mortality, the single included RCT [27] was evaluated as having a low risk of bias, whereas the prospective cohort studies [24,25,26] exhibited several significant quality limitations (Tables S4 and S5). Methodological reporting across the cohort studies [24,25,26] was generally poor: only one study clearly and adequately described the baseline characteristics of the study population [24], and none specified initial participation rates or provided a statistical power calculation based on sample size. Furthermore, only one study evaluated Se exposure as a continuous variable [26], and only one performed serial Se measurements [25]. Crucially, none of the studies reported whether outcome assessors were blinded to participant exposure status, nor did they explicitly detail the adjustment for confounding variables in their statistical analyses, thereby introducing a high risk of bias. In one study, the timeframe for outcome assessment could not be safely judged as adequate [26]. Finally, the study by Katsaka et al. (possibly because it was published as a conference poster) exhibited further limitations, failing to clearly define eligibility criteria, validate exposure and outcome assessment methods, or report loss-to-follow-up rates [25].

The evidence regarding overall neonatal morbidity risk was characterized by contrasting methodological quality. The RCT by Karamali et al. was classified as having a low risk of bias [29] (Table S4). Conversely, the prospective cohort study by Bakhtadze et al. lacked clarity regarding initial participation rates, eligibility criteria for the study population, the validity of outcome measurement methods, loss-to-follow-up rates, and whether outcome assessors were blinded to participant exposure status. Furthermore, the authors did not detail the baseline characteristics of the included population, provide a justification for the sample size, perform serial Se measurements, or adjust for potential confounding variables in their statistical analysis [28] (Table S5). To be noted, the aforementioned limitations are partially attributable to its publication as a conference poster [28].

The two studies evaluating both neonatal mortality and morbidity exhibited markedly different quality profiles. The RCT by Boskabadi et al. was judged to be at high risk, due to concerns regarding the randomization process, missing outcome data, and selective outcome reporting [30] (Table S4). Conversely, the prospective cohort study by Modzelewska et al. demonstrated stronger methodology [31] (Table S5). However, certain limitations were identified: the authors did not provide a statistical power estimation, failed to conduct serial Se exposure measurements, and reported an initial participation rate below 50%. Additionally, it remained unclear whether outcome assessors were blinded to the exposure status of the participants [31].

The subgroup of prospective cohort studies examining neonatal pulmonary disease demonstrated moderate to high methodological quality [33,34,35] (Table S5). However, specific reporting deficiencies were noted across the papers: one study failed to clearly describe the baseline characteristics of the study population [34], and only one of the three studies explicitly specified the initial participation rate, provided a sample size justification, and adjusted for potential confounding variables in the statistical analysis [35]. Furthermore, two studies did not clearly report whether the outcome assessors were blinded to participant exposure status [33,35], and none performed serial Se measurements. Additionally, the case–control study by Falciglia et al. did not justify the sample size selection, employ concurrent controls, or adjust for potential confounders in the statistical analysis [32] (Table S6). It also remained unspecified whether the selected cases were representative of the target population or if they were randomly selected from the eligible case pool [32].

Regarding infectious causes of mortality and morbidity, the relevant prospective cohort studies demonstrated moderate methodological quality [36,37,38] (Table S5). Several consistent reporting and design limitations were observed across this subgroup: none of the studies clearly reported initial participation rates, provided a justification for sample size, or performed serial Se measurements. Furthermore, it remained unclear in all included studies whether outcome assessors were blinded to participant exposure status, and only a single study explicitly specified the loss-to-follow-up rate [36]. Finally, Zhang et al. failed to adjust for potential confounding variables in their statistical analysis [38].

The prospective cohort studies examining neurobehavioral development demonstrated moderate to high methodological quality [39,40,41,42] (Table S5). However, several systematic limitations were identified: two out of the four studies did not report whether outcome assessors were blinded to participant exposure status [40,42], and three failed to specify initial participation rates [39,40,42]. Crucially, none of the studies provided a sample size justification or included a statistical power estimation. None performed serial Se measurements or established an adequate timeframe between exposure and outcome assessment to support a causal relationship. Across all included studies, Se levels were measured exclusively at the time of delivery, while neurobehavioral development was assessed between 24 h and two weeks postpartum.

### 3.8. Certainty of the Evidence

The GRADE certainty of evidence varied across the evaluated neonatal outcomes, ranging from moderate to very low. Evidence was graded as moderate for combined mortality and morbidity. Specifically, the two articles assessing combined mortality and morbidity were downgraded solely for risk of bias because one of the two studies (an RCT) was considered to be at high risk [30,31].

A low level of certainty was assigned to the evidence for all-cause neonatal or perinatal mortality, infectious causes of mortality and morbidity, and neurobehavioral development. The four articles evaluating all-cause neonatal or perinatal mortality were downgraded due to imprecision from small sample sizes and a high risk of bias, as none specified the adjustment for possible confounders in their statistical analysis [24,25,26,27]. The three articles examining infectious causes were also categorized as low certainty, losing one point in the imprecision domain and one point in the indirectness domain, due to the evaluation of clinically distinct outcomes [36,37,38]. Lastly, the four articles in the neurobehavioral development group were downgraded to low certainty due to inconsistency and indirectness, because all exposure samples were collected at the time of delivery [39,40,41,42].

The evidence was evaluated as very low certainty for both all-cause morbidity and pulmonary-related mortality and morbidity. The two articles examining all-cause morbidity were downgraded across four domains: risk of bias (with one study considered at high risk), inconsistency due to statistically significant results of opposite directions, imprecision from small sample sizes, and possible dissemination bias [28,29]. Finally, the four articles examining neonatal mortality and morbidity due to pulmonary conditions were downgraded to very low certainty across three domains [32,33,34,35], driven by a high risk of bias (three out of four articles), indirectness from measuring Se levels around the time of delivery, and imprecision, as three out of four studies featured small sample sizes.

## 4. Discussion

This systematic review synthesized the clinical and epidemiological evidence evaluating the impact of maternal Se exposure during pregnancy on various neonatal outcomes, revealing substantial variations in both methodological certainty and clinical efficacy. Overall, the available literature does not support an inverse association between antenatal Se exposure and all-cause neonatal or perinatal mortality, neurobehavioral development, or composite neonatal mortality and morbidity, with the latter even demonstrating a weak, statistically unstable signal toward potential positive association in a highly powered cohort. Similarly, low-certainty prospective evidence indicates that maternal Se status showed no clear association with vertical viral transmission or early-life bacterial sepsis susceptibility. Conversely, inverse relationships were observed only for neonatal pulmonary morbidity, where epidemiological indications suggest lower risks of respiratory conditions, and in a single low-risk randomized controlled trial demonstrating reduced neonatal morbidity in pregnancies affected by gestational diabetes. However, these findings are highly contrasted by observational data linking elevated maternal Se to the composite outcome of increased RDS or NICU admission risk. Overall, the evidence remains heavily constrained by moderate to very low certainty across several domains—predominantly driven by small sample sizes, a lack of confounding adjustments, and indirectness from exposure measurements taken exclusively at delivery, highlighting the need for rigorous, standardized future investigations.

### 4.1. Public Health Relevance of Neonatal Mortality and Morbidity

Neonatal mortality, defined as infant death occurring during the first 28 days of life, remains a critical public health issue characterized by considerable geographic and socioeconomic disparities, both between and within countries. Over the past decades, several advancements have been recorded, owing primarily to superior antenatal surveillance and care, expanded general access to healthcare, and clinical improvements in newborn resuscitation and management. In 2022, approximately 2.3 million neonates died globally, accounting for 47% of all deaths among children under five years of age; nonetheless, this figure represents a substantial improvement compared to the 5.0 million deaths recorded in 1990 [1]. Yet, more than 60 nations are currently projected to fall short of achieving the 2030 World Health Organization (WHO) Sustainable Development Goal target of ≤12 deaths per 1000 live births [1]. With respect to geographic variation, sub-Saharan Africa, followed by Central and Southern Asia, exhibits the highest neonatal mortality rates, with both regions exceeding 20 deaths per 1000 live births [46]. Conversely, Australia and New Zealand, Europe, Eastern Asia, and Northern America consistently record fewer than 4 deaths per 1000 live births [46]. Notably, considerable intra-regional variations do exist within these broad boundaries. For instance, within Europe in 2023, mortality rates ranged from 0.5 in Estonia to 5 deaths per 1000 live births in Moldova, representing a profound ten-fold difference across the continent [47].

These high rates of neonatal morbidity and mortality are fundamentally driven by the heightened biological susceptibility of the neonate during the critical physiological transition to extrauterine life. The principal direct contributors to early mortality are prematurity and its associated complications, birth asphyxia, and perinatal infections [2,48]. Apart from these primary causes, low birth weight, multiple gestations, cesarean or instrumental deliveries, inadequate antenatal care, young maternal age, low maternal educational attainment, low Apgar scores, and major congenital malformations are all established risk factors for neonatal death [2,5,48]. With regard to prematurity, infants frequently face long-term sequelae even after surviving the initial neonatal period. These chronic complications span respiratory, gastrointestinal, and immunological systems, and map onto the central nervous system, manifesting as long-term neurobehavioral problems that further emphasize the enduring public health implications of neonatal morbidity [6].

With respect to the neurobehavioral development of preterm and critically ill neonates, several distinct elements interact with the structural immaturity of the central nervous system [49]. These include maternal sociodemographic factors, such as educational level, socioeconomic status, and antenatal smoking; infant characteristics, such as male sex and low Apgar scores [49]; and neonatal intensive care unit (NICU) environment, including noxious sensory stimuli, parental interaction barriers, and caregiving behaviors [50]. The global increase in NICU admissions has catalyzed research into the neurodevelopmental impact of the NICU environment itself. Because sensory and environmental stimuli are highly important in shaping motor, sensory, behavioral, and cognitive neurological pathways, novel, neuro-protective NICU frameworks have been proposed, such as the Newborn Individualized Developmental Care and Assessment Program (NIDCAP) and Kangaroo Mother Care [50].

Given the profound public health importance of mitigating neonatal mortality, morbidity, and adverse early-life environments, identifying modifiable maternal exposures during pregnancy represents an essential research priority. Targeted gestational exposures, including maternal Se status, hold significant potential to optimize the intrauterine environment through lifestyle modifications, nutritional counseling, or minimally invasive interventional strategies.

### 4.2. Pathophysiological Mechanisms

Se is an essential trace element exerting its physiological effects primarily via selenocysteine incorporation into 25 distinct selenoproteins [7]. Glutathione peroxidases (GPx), the most abundant selenoproteins, reduce hydroperoxides and free radicals via glutathione oxidation to protect tissues from oxidative damage [7,9]. Thioredoxin reductases (TrxR) regulate cell growth, DNA synthesis, and cellular redox homeostasis [7,8], while iodothyronine deiodinases (DIOs) peripherally convert prohormone thyroxine (T4) into active triiodothyronine (T3), supporting thyroid and metabolic function [8,9]. Additional key selenoproteins include selenoproteins S (SELENOS), N (SELENON), and P (SELENOP), with SELENOP serving as the primary plasma Se transport protein and functional status biomarker [8].

Animal models underscore the critical role of Se in fetal brain development, where fetal brain Se concentrations are preferentially maintained during maternal deficiency [39,51]. In mice, targeted Gpx4 deletion induces postnatal ataxic gait and hyperexcitability [52], while TrxR1 ablation precipitates profound growth retardation and movement disorders [53,54]. Furthermore, in mice deleting SELENOP, which facilitates Se uptake across the blood–brain barrier, causes severe neurological dysfunction, including spasticity and spontaneous seizures [8,55]. Homeostatic importance is further underscored by the narrow therapeutic window of Se, where excess also impairs neurobehavioral development across animal models. Maternal Se supplementation in pregnant mice induced offspring sensorimotor reflex and learning deficits [56]. Similarly, selenomethionine (SeMet) exposure in female zebrafish caused offspring stress, altered social behavior, and serotonergic dysregulation [57]. In embryo zebrafish, SeMet exposure at 5 and 10 μg/L elevated mortality and impaired reflexive behaviors [58].

A broad body of evidence points toward a sensitivity of the central nervous system to Se status in humans. Altered Se has been linked to cognitive decline in older adults [8]. In children, higher maternal Se during pregnancy has been linked to compromised cognitive development [51,59], while lower prenatal Se has been linked to compromised language and motor development [40,60]. Importantly, excessive Se may induce neurotoxic effects, such as optic nerve demyelination in children and adverse behavioral phenotypes or depressed passive muscle tone at birth [39,40,61]. Regarding the neonatal period, which has been examined in our systematic review, indirect indications of similar non-linear, U-shaped dynamics have been observed. Antenatal Se supplementation reduced overall neonatal morbidity in an Iranian GDM trial, hypothesized to reflect high baseline oxidative stress [29], whereas elevated native Se in a Georgian cohort was associated with increased RDS risk, speculatively linked to pro-oxidant mechanisms [28]. However, as primary studies did not measure baseline oxidative status, these explanations remain speculative.

From a mechanistic standpoint, gestational Se may exert multi-faceted protective effects on the developing central nervous system through direct selenoprotein action, thyroid hormone regulation, and mitigation of heavy metal toxicity. SELENOP facilitates Se transport into the fetal brain [8], while TrxR1 is essential for cerebellar morphogenesis [62]. GPx enzymes protect neural tissue from oxidative stress and lipid peroxidation while modulating redox-sensitive transcription factors [53]. Specifically, neuronal and astrocytic GPx1 reduces hydrogen peroxide (H2O2) and organic hydroperoxides, whereas GPx4 protects neuronal membrane lipids across subcellular compartments [62]. Downstream effects of maternal Se status on neonatal neurobehavioral outcomes may also be indirectly mediated by maternal thyroid function, which strongly dictates offspring cognitive outcomes [63,64]. Local tissue availability of biologically active thyroid hormones within developing fetal brain cells is tightly regulated by DIOs, enzymes whose catalytic capacity is fundamentally Se-dependent [63]. Optimal gestational Se levels may also mitigate heavy metal-induced neurotoxicity from environmental elements such as cadmium (Cd) and manganese (Mn). Adequate Se attenuates early neurodevelopmental toxicity via competitive antagonism and selenoprotein-mediated upregulation of antioxidant pathways, thereby neutralizing heavy metal-induced oxidative stress in fetal tissues [42,65].

Beyond the central nervous system, Se is critical for other rapidly developing systems. Prematurity complications, including RDS, BPD, retinopathy of prematurity (ROP), and intraventricular hemorrhage (IVH), are fundamentally driven by oxidative stress, a process Se counteracts [66]. Clinically, Se deficiency correlates with compromised respiratory outcomes in preterm infants [67]. Oxidative stress participates in RDS and BPD pathophysiology and is, further, exacerbated by necessary neonatal interventions, including oxygen therapy and mechanical ventilation [68]. Preterm infants are particularly vulnerable due to immature antioxidant systems coupled with low baseline Se, caused by premature interruption of transplacental Se transfer, which occurs predominantly during the third trimester alongside hepatic accumulation from the 20th week onward [67,68]. Post-delivery, ongoing oxidative stress further depletes these limited Se reserves through accelerated GPx enzyme synthesis [69].

Se is also critical for maintaining proper placental function. In mice, Se deficiency induces placental oxidative stress, inhibits trophoblast proliferation, disrupts autophagy, and triggers apoptosis within placental tissue [70]. Furthermore, maternal Se deficiency in mice causes placental necrosis foci and calcium deposits, suppresses nutrient transporter expression, immune response activation, and disrupts progesterone biosynthesis, a steroid hormone essential for pregnancy maintenance [71]. Correspondingly, treating first-trimester human placental tissue with sodium selenite significantly increases cell proliferation while reducing apoptosis and DNA damage, irrespective of baseline oxidative stress [72].

Lastly, Se status may also influence fetal and neonatal health by regulating systemic and cellular iron homeostasis. Preterm infants exhibit elevated free iron compounds, which catalyze Fenton reactions that generate toxic hydroxyl radicals [68]. Unmitigated oxidative stress and dysregulated iron homeostasis drive ferroptosis, an iron-dependent regulated cell death pathway characterized by mitochondrial shrinkage, cristae loss, and lethal lipid ROS accumulation [73]. In neonatal pathology, ferroptosis is linked to neural injury and hypoxic–ischemic encephalopathy (HIE) [73]. Mechanistically, Se may mitigate ferroptosis primarily through GPx4, the principal selenoprotein responsible for neutralizing lipid hydroperoxides and preventing iron-mediated lipid peroxidation. Nevertheless, a profound discrepancy remains between compelling biomolecular mechanisms, robust animal data, and clinical reality. Despite biological plausibility across selenoprotein pathways, thyroid regulation, heavy-metal antagonism, and ferroptosis mitigation, synthesized empirical evidence indicates that maternal Se status does not translate into widespread clinical benefits for neonates. With isolated exceptions, namely an inverse association with neonatal pulmonary morbidity across reviewed studies and an inverse association with all-cause morbidity in a single GDM trial, most outcomes, including all-cause mortality, infectious susceptibility, and neurobehavior, showed no clear association with maternal Se exposure. These findings suggest preclinical protective pathways may be masked by complex clinical confounders in human populations or already operate at functional saturation under physiological conditions.

### 4.3. Strengths, Limitations, and Future Research Directions

This systematic review sought to comprehensively evaluate the impact of gestational Se exposure on neonatal mortality, morbidity, and neurodevelopment. A primary strength of this study lies in its adherence to a pre-planned methodology and execution in accordance with the PRISMA guidelines, ensuring transparency in data extraction and reporting. Furthermore, to ensure the validity and reliability of our findings, we utilized validated tools to systematically evaluate the risk of bias within the included studies and applied standardized procedures to assess the certainty of the body of evidence for each outcome subgroup. Lastly, we assessed the impact of including two conference abstracts, confirming that the core findings and conclusions of this systematic review remained materially unchanged regardless of their inclusion.

Nonetheless, several limitations must be acknowledged. Methodological and clinical heterogeneity across the included literature precluded the poolability of data for a formal meta-analysis. This heterogeneity stems primarily from the clinical heterogeneity of gestational Se exposure definitions across included studies and the broad, “umbrella” definition of neonatal morbidity. Biological matrices (maternal serum/plasma, full blood, cord blood, and placenta) and supplementation regimes were combined within outcome domains due to the small number of available studies per endpoint (2–4 studies per outcome group). While further disaggregation by specific matrix was not feasible without precluding synthesis altogether, this matrix variation may introduce heterogeneity and obscure potential matrix-specific exposure-outcome relationships. Regarding outcome definition variability, the reviewed studies evaluated highly disparate health conditions; even within the same outcome subgroup, studies frequently differed in their specific outcome definitions or utilized different measurement scales to assess the same clinical endpoint. In addition, several primary studies utilized broad composite outcomes for all-cause neonatal morbidity (combining diverse conditions such as respiratory distress, sepsis, and hyperbilirubinemia) rather than disaggregated endpoints. As a result, sub-analyses by specific disease pathology were not feasible, which should be considered when interpreting condition-specific risk associations. Another substantial constraint is the relatively small number of eligible studies and their correspondingly small sample sizes. Additionally, significant variations were observed regarding the statistical methods applied and the timing of exposure assessments. Crucially, many studies measured Se levels exclusively around the time of delivery, which obscures the temporal sequence and limits the ability to establish a direct causal relationship between prenatal exposure and neonatal outcomes. Maternal circulating Se concentrations fluctuate dynamically throughout gestation due to physiological plasma volume expansion, increased fetal uptake, active placental transport, and altered metabolic demands. Thus, peripartum concentrations may poorly reflect exposure profiles during earlier, critical neurodevelopmental or organogenetic windows. Additionally, acute perinatal events and clinical complications may acutely alter trace element kinetics at delivery, raising the possibility of reverse causality. The lack of multivariable adjustment for major confounding variables in nearly half of the included primary studies (9 out of 19) is also recognized as an additional critical limitation. This unadjusted design was particularly frequent among studies evaluating all-cause neonatal or perinatal mortality, all-cause neonatal morbidity, combined neonatal mortality and morbidity, and neonatal pulmonary conditions. However, factors such as maternal smoking and socioeconomic position can exert confounding effects. Smoking induces high oxidative demand that depletes maternal Se status while independently elevating neonatal risks, whereas low socioeconomic position often correlates with restricted dietary Se intake, poorer nutritional quality, and adverse perinatal conditions. Without adequate control for these structural and lifestyle factors, residual confounding may either mask subtle biological effects or introduce spurious associations. Finally, despite extensive retrieval efforts, including contacting corresponding authors and leveraging multi-institutional international networks, the full text for 26 records could not be obtained. None of these records reported neonatal mortality, morbidity, or neurodevelopmental outcomes in their abstracts. However, reporting of these endpoints within the full text cannot be definitively excluded. Thus, partial information retrieval, due to 26 unavailable full-texts and 36 reports published in languages other than English, represents a minor risk of selection bias. Additionally, while all methodological steps of the SeduP project were a priori designed, a formal protocol for this systematic review was not prepared and prospectively registered in a public database.

Despite these limitations, this systematic analysis successfully identifies critical knowledge gaps to guide future research. To generate findings that are generalizable to diverse pregnant populations and translatable into future nutritional guidelines, large-scale, adequately powered studies are required. Future investigations should adopt standardized statistical approaches that simultaneously address primary mortality and morbidity endpoints, thereby ensuring data are suitable for direct comparison and meta-analytical synthesis. Moreover, to establish exposure trajectories and clear causal pathways, future studies should standardize the timing and matrix of Se exposure assessment. Ideally, sampling of the maternal compartment should occur at multiple gestational trimesters, with emphasis on the relevant neurodevelopmental and organogenetic windows and the latter stages of gestation that coincide with accelerated transplacental mother-to-fetus Se transport, rather than at the exact time of delivery. Moving forward, emerging research must focus on enhancing the clinical relevance and applicability of its findings, actively overcoming the challenge of heterogeneity to provide actionable evidence for clinicians.

## 5. Conclusions

In conclusion, while robust preclinical and mechanistic models provide a compelling biological plausibility for Se in supporting fetal development and optimizing neonatal outcomes, these theoretical benefits do not currently translate into widespread, clinically demonstrable protection for human neonates. This systematic review indicates that maternal Se status during pregnancy shows no clear association with the risk of all-cause neonatal morbidity and mortality, vertical viral transmission and early-life bacterial sepsis, or neurobehavioral deficits. Instead, clinical signals remain highly localized and conflicting, evidenced by very-low-certainty data suggesting potential protection against neonatal pulmonary morbidity alongside isolated data linking maternal Se excess to an increased risk of RDS and NICU admission. Ultimately, the existing body of evidence is heavily constrained by moderate to very low methodological certainty, characterized by small sample sizes, a lack of confounding adjustments, and critical exposure assessment timing limitations. Given these widespread unadjusted analyses and cross-sectional sampling designs, all reported findings, including null associations, must be interpreted with appropriate clinical caution. Overall, current evidence is insufficient to demonstrate consistent benefit or harm of maternal Se exposure during pregnancy, with substantial uncertainty caused by heterogeneous exposure measures, sparse data, residual confounding, and moderate to very low certainty. To bridge this gap between biological theory and clinical application, future research must prioritize large-scale, prospectively registered cohorts that utilize standardized biological sampling matrices throughout gestation.

## Figures and Tables

**Table 1:** The search strategy in PubMed.

(“pregnancy” [MeSH Terms] OR “pregnan * ”[Title/Abstract] OR “gestation” [Title/Abstract] OR “prenatal” [Title/Abstract] OR “intrauterine” [Title/Abstract] OR “in utero” [Title/Abstract] OR “Perinatal” [Title/Abstract] OR “Postnatal” [Title/Abstract])
AND
(“Selenium” [MeSH Terms] OR “selenium” [Title/Abstract])
NOT
(“animals” [MeSH Terms] NOT “humans” [MeSH Terms])

* Indicates truncation used to capture multiple word variants sharing the same root.

## Data Availability

No new data were created or analyzed in this study. Data sharing is not applicable to this article.

## Supplementary Materials

The following supporting information can be downloaded at: https://www.mdpi.com/article/10.3390/jcm15166255/s1; Table S1: The search strategy in Embase; Table S2: The search strategy in Cochrane Library; Table S3: Summary of findings Table of the reviewed studies; Table S4: Quality appraisal of the included randomized control trials; Table S5: Quality appraisal of the included cohort studies; Table S6: Quality appraisal of the included case–control studies; Table S7: The PRISMA 2020 checklist for abstracts; Table S8: The PRISMA 2020 checklist.

## Abbreviations

The following abbreviations are used in this manuscript:

Se	Selenium
NICU	Neonatal intensive care unit
RDS	Respiratory distress syndrome
SEduP	Selenium exposure during pregnancy
MeSH	Medical Subject Headings
CMV	Cytomegalovirus
HIV	Human immunodeficiency virus
GRADE	Grading of Recommendations Assessment, Development, and Evaluation
RCT	Randomized controlled trial
PRISMA	Preferred Reporting Items for Systematic Reviews and Meta-Analyses
GDM	Gestational diabetes mellitus
CPAP	Continuous positive airway pressure
BPD	Bronchopulmonary dysplasia
NBNA	Neonatal behavioral neurological assessment
NNNS	Neonatal Intensive Care Unit Network Neurobehavioral Scale
NOS	Neurologic Optimality Score
WHO	World Health Organization
NIDCAP	Newborn Individualized Developmental Care and Assessment Program
GPx	Glutathione peroxidase
TrxR	Thioredoxin reductases
DIO	Iodothyronine deiodinases
T4	Thyroxine
T3	Triiodothyronine
SELENOS	Selenoproteins S
SELENON	Selenoproteins N
SELENOP	Selenoproteins P
SeMet	Selenomethionine
ROS	Reactive oxygen species
Cd	Cadmium
Mn	Manganese
ROP	Retinopathy of prematurity
IVH	Intraventricular hemorrhage
HIE	Hypoxic–ischemic encephalopathy
